# γ-H2AX promotes hepatocellular carcinoma angiogenesis via EGFR/HIF-1α/VEGF pathways under hypoxic condition

**DOI:** 10.18632/oncotarget.2942

**Published:** 2014-12-10

**Authors:** Heng Xiao, Rongliang Tong, Chaofeng Ding, Zhen Lv, Chengli Du, Chuanhui Peng, Shaobing Cheng, Haiyang Xie, Lin Zhou, Jian Wu, Shusen Zheng

**Affiliations:** ^1^ Division of Hepatobiliary and Pancreatic Surgery, Department of Surgery, First Affiliated Hospital, Zhejiang University School of Medicine, Hangzhou, China; ^2^ Key Lab of Combined Multi-Organ Transplantation, Ministry of Public Health, Hangzhou, China

**Keywords:** hepatocellular carcinoma (HCC), γ-H2AX, angiogenesis, vascular endothelial growth factor (*VEGF*), epidermal growth factor receptor(EGFR), hypoxia inducible factor 1α (HIF-1α)

## Abstract

Hepatocellular carcinoma (HCC) is one of the most deadly cancers. Using mRNA microarray analysis, we found that H2AX decreased under hypoxic conditions. Hypoxia is an important physiological and pathological stress that induces H2AX phosphorylation (γ-H2AX), but the regulatory mechanism of γ-H2AX remains elusive in the progress of HCC. We report here that increased γ-H2AX expression in HCC is associated with tumor size, vascular invasion, TNM stage and reduced survival rate after liver transplantation (LT). γ-H2AX knockdown was able to effectively inhibit VEGF expression *in vitro* and tumorigenicity and angiogenesis of HCC *in vivo*. The mechanism of γ-H2AX on the angiogenic activity of HCC might go through EGFR/HIF-1α/VEGF pathways under hypoxic conditions. Combined γ-H2AX, HIF-1α and EGFR has better prognostic value for HCC after LT. This study suggests that γ-H2AX is associated with angiogenesis of HCC and γ-H2AX or a combination of γ-H2AX/EGFR/HIF-1α is a novel marker in the prognosis of HCC after LT and a potential therapeutic target.

## INTRODUCTION

Hepatocellular carcinoma (HCC), a highly vascular tumor, is the third leading cause of cancer deaths worldwide, and the second in China [1-3]. In view of the insufficiency of existing therapies for eradicating this tumor and the high frequency of its recurrence, the prognosis of HCC patient remains discouraging. Hence, we found it important to illustrate the molecular mechanisms of angiogenesis of HCC, and to establish the identity of new targets for therapeutic approach which can improve the prognosis of HCC patients.

H2A histone family member X (H2AX or H2AFX) and its phosphorylated C-terminal (Sre residues 139-140, γ-H2AX) are crucial in DNA damage response and the mediation of DNA repair [4-6]. As it is known, hypoxia is an important physiological and pathological stress that induces DNA damage response and γ-H2AX expression [7-10]. It is, however, widely believed that hypoxia is a major force for neovascularisation due to expression of hypoxia-inducible factor(HIF) that helps accelerate endothelial cell proliferation and migration [11, 12]. Previous researches have demonstrated that hypoxia can induce the expression of γ-H2AX through the DNA damage response [9, 13]. Recently, some studies have suggested that hypoxia-induced γ-H2AX are dramatically present in the proliferating endothelial cells. While the H2AX knock-down or knock-out under hypoxic conditions, the hypoxia-driven retina angiogenesis was obviously weakened [14-16]. Currently, transhepatic arterial chemotherapy embolization and local stereotactic radiotherapy play an imperative role in the treatment of HCC. The mechanism behind these treatments is the induction of cleavage in the DNA double strands which consequently promotes tumor cell apoptosis and necrosis, however, abnormal angiogenesis remarkably increases with an ascending residual tumor cells' malignant degree [17-23]. Therefore, γ-H2AX may play a complex role in HCC angiogenesis.

In this study, the contribution of γ-H2AX to HCC angiogenesis was investigated in the context of hypoxia. Results presented here suggest that γ-H2AX is an indispensable way besides HIF in promoting HCC angiogenesis via modulation of γ-H2AX/EGFR/HIF-1α/VEGF signaling. We propose that the combination of γ-H2AX/EGFR/HIF-1α is a new potential target for treatment of HCC.

## RESULTS

### Effect of hypoxia on mRNA expression in HCC cells

**Figure 1:**
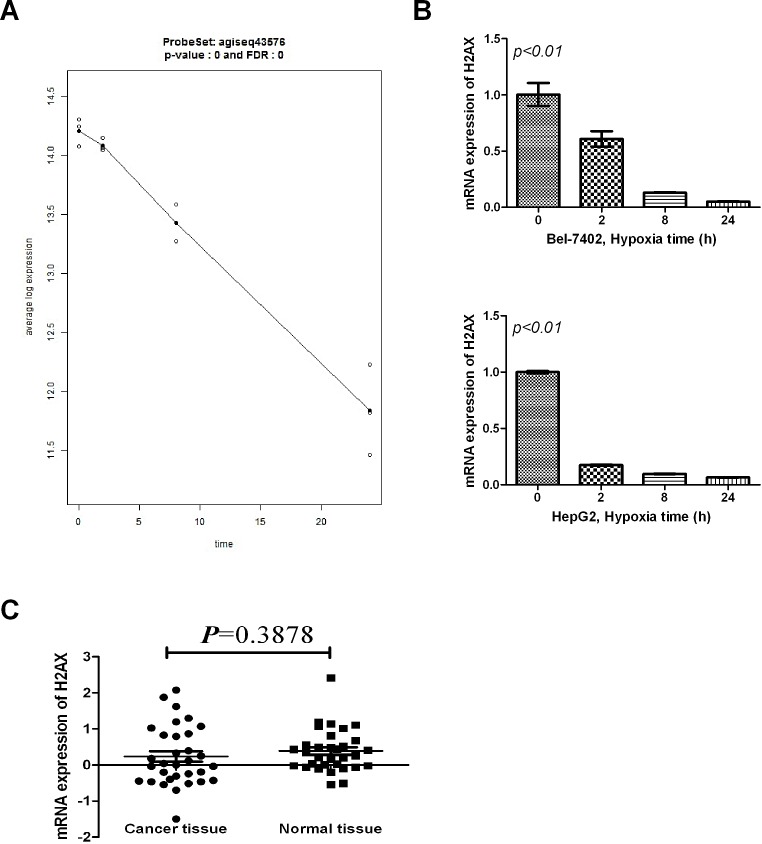
Effect of hypoxia on H2AX mRNA expression in HCC cells (A) The line plot of H2AX mRNA detected by microarray analysis. (B) Levels of H2AX expression in normoxic and hypoxic conditions (two cell lines-HepG2 and Bel-7402) were determined using RT-PCR. (C) Expression levels of H2AX in 32 samples of HCC tissue were analyzed using RT-PCR.

We used mRNA array hybridization to compare the mRNA expression profiles in normoxic and hypoxic cells. Data analysis selected many mRNA with expression that differed between normoxic and hypoxic cells at 2, 8, 24hours. Among these mRNAs, H2AX was the primary focused (Fig. 1A). We confirmed H2AX expression change using RT-PCR, which showed H2AX to be significantly down-regulated in hypoxia culture (Fig. 1B). To investigate the level of H2AX in HCC patients, RT-PCR was used to estimate the expression of H2AX in 32 samples of HCC tissue. Unfortunately, tissue level of H2AX expression did not differ (Fig. 1C). However, its C-terminal phosphorylation (Sre residues 139-140, γ-H2AX) are crucial in the DNA damage response under hypoxic condition, and hence, we estimated the level of γ-H2AX in HCC tissues by immunohistochemistry.

### Up-regulation of γ-H2AX correlates with recurrence of HCC patients

**Figure 2:**
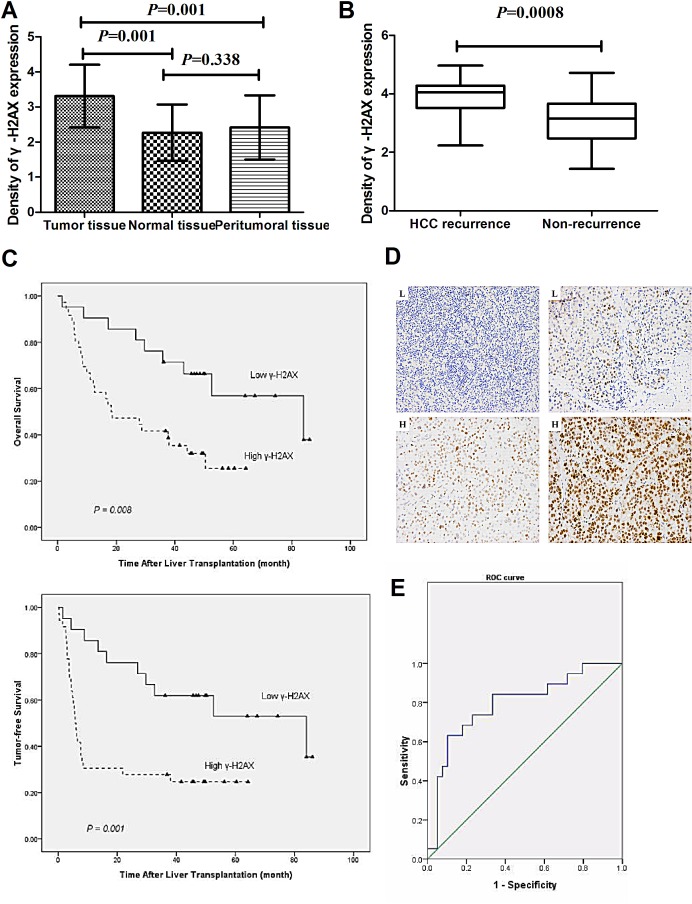
Increased levels of γ-H2AX indicate worsening prognosis and recurrence/metastasis of HCC (A) Protein levels of γ-H2AX were determined in 57 samples of HCC, 37 samples of peritumoral tissues and 17 samples of normal tissues samples. (B) Protein levels of γ-H2AX in tumor tissues of patients with post-LT HCC recurrence (n = 24) in comparison to patients with no-recurrence (n = 33). The unit of scale (y-axis of the (A) and (B)) presents the staining density. (C) The tumor-free and over-all survival rates of 57 patients with HCC post LT were compared between the low-γ-H2AX and high-γ-H2AX groups. (D) HCC samples in a tissue microarray were immunostained with a monoclonal anti-γ-H2AX antibody. Representative low-γ-H2AX (L) and high-γ-H2AX expression (H) samples are also shown (×200). (E) ROC curve of γ-H2AX level was used to predict tumor recurrence and non-recurrence after liver transplant.

In this study, the levels of γ-H2AX in 57 samples of HCC, 37 samples of peritumoral tissue and 17 samples of normal tissue were examined by immunohistochemistry. The results showed that γ-H2AX levels (Fig. 2A) were higher in tumor tissues of patients than peritumoral tissues (p = 0.001) and normal tissues (p = 0.001). And the results showed that γ-H2AX levels were higher in tumor tissues of patients with post-LT HCC recurrence (n = 24) in comparison to patients with no-recurrence (n = 33) (p < 0.001) (Fig. 2B). To investigate the clinicopathologic features of γ-H2AX in HCC and to determine whether γ-H2AX expression in HCC was associated with tumor-free survival and overall post-transplanted survival, all 57 patients with HCC after LT were divided into two groups: the high-expression group (n = 36) and low-expression group (n = 21). The results showed that tumor size, vascular invasion and TNM stage showed significant differences (p < 0.05) (Table 1). Kaplan–Meier analysis revealed that HCC tissues with high expression of γ-H2AX had either worse overall survival (p = 0.002) or shorter tumor-free survival (p < 0.001) (Fig. 2C, D). The 1, 3 and 5 year overall survival rates among patients with high-γ-H2AX were 63.9%, 41.7% and 25.5%, respectively, whereas the rates in the patients with low-γ-H2AX were 90.5%, 71.4% and 59.6 %. Moreover, the 1, 3 and 5 years tumor-free survival after LT were much worse for high-γ-H2AX than low-γ-H2AX expression group (Table 2). ROC curve of γ-H2AX level was used to predict tumor recurrence and non-recurrence after LT. When considering a cutoff point of 0.79, sensitivity and specificity were 79.6% and50.6 % respectively with an AUC value of 0.79 (Fig. 2E). Thus, γ-H2AX is a valuable predicting factor for recurrence and survival in patients with HCC after LT.

**Table 1:** Relationship between γ-H2AX expression and clinicopathologic features

	γ-H2AX Density		
Variable	low-γ-H2AX	high-γ-H2AX	*P* value
In general			
Normal tissue	14	3	
Peritumoral tissue	25	12	
Tumor tissue	21	36	0.001, 0.003
Sex			
Male	19	33	0.602
Female	2	3	
Age(years)			
≦50	5	21	0.013
>50	16	15	
Tumor size(cm)			
≦5	18	20	0.022
>5	3	16	
AFP(ng/ml)			
≦400	5	13	0.212
>400	16	23	
HBsAg			
Positive	17	30	0.525
Negative	4	6	
Anti-HCV			
Positive	1	1	0.597
Negative	20	35	
Vascular invasion			
Yes	3	19	0.005
No	18	17	
TNM stage			
I-II	16	10	<0.001
III-IV	5	26	

**Table 2:** Relationship between γ-H2AX expression and survival rate

	γ-H2AX Density		
Survival Measurement	low-γ-H2AX	high-γ-H2AX	*P* value
1-year overall survival (%)	90.5±6.4	63.9±8.0	0.002
3-year overall survival (%)	71.4±9.9	41.7±8.2	
5-year overall survival (%)	56.9±12.5	25.5±8.6	
1-year tumor-free survival (%)	85.7±7.6	30.6±7.7	<0.001
3-year tumor-free survival (%)	61.9±10.6	27.8±7.5	
5-year tumor-free survival (%)	53.1±12.2	24.7±7.2	

### γ-H2AX correlates with VEGF expression *in vitro* and H2AX knockdown inhibits tumorigenicity and angiogenesis of HCC *in vivo*

**Figure 3:**
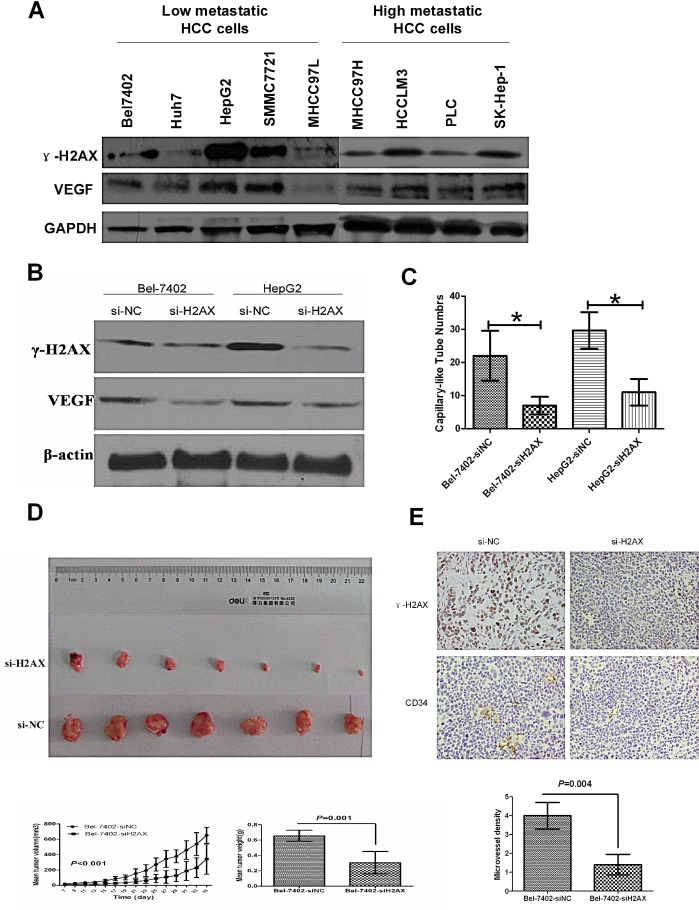
Down-regulation of γ-H2AX inhibits angiogenesis and tumorigenicity of HCC cells (A) γ-H2AX protein expression was determined by western blot in 9 HCC cell lines. GAPDH was used as a positive control. (B) Effect of H2AX knockdown on VEGF expression was measured by western blot. (C) Effect of γ-H2AX down-regulation on capillary-like tube structure formation was measured by HUVECs *in vitro*. (D) *In vivo* tumorigenesis assay of tumor size and weight after transfected HCC cells were injected subcutaneously into nude mice. (E) Immunohistochemistry analysis of γ-H2AX and CD34 in nude mice tumor tissues. The microvessel density was calculated in tumors from each group.

To prove the significance of the above clinical data, we examined the γ-H2AX and VEGF expressions in nine of the low and high metastatic HCC cell lines. We found that VEGF showed the same change with the level of γ-H2AX expression (Fig.3A). To determine the relationship between γ-H2AX and VEGF, we established H2AX knock-down cell lines. For stable knockdown of H2AX, Bel-7402 and HepG2 were transfected with a GFP-lentiviral vector (Scr-siRNA/GFP) which was used as a negative control (NC). The effective H2AX knockdown inhibited VEGF protein expression (Fig. 3B). Then we examined the angiogenesis of HUVECs by tube formation assay with conditional culture medium from H2AX knockdown cells or control cells, the results showed that the inhibition of H2AX significantly suppressed capillary-like tube structure formation by HUVECs (Fig.3C). We further examined the effect of γ-H2AX on HCC growth and angiogenesis in nude mice. H2AX-siRNA-transfected SMMC-7402 cells and negative control group cells (NC) were injected subcutaneously into nude mice (7 animals per group). All mice were sacrificed, and the tumors were dissected at end of the experiment. Compared to the NC group, H2AX knockdown resulted in a significant decrease in tumor size and weight (P<0.001) (Fig. 3D). The tumors were stained with γ-H2AX, and CD34. The expression of γ-H2AX and CD34 were observed to decrease in H2AX knockdown-injected tumors as compared with NC-injected mice (Fig. 3E). Together, these results reveal the biological function of γ-H2AX expression in HCC angiogenesis.

### γ-H2AX is essential for HIF-1α-mediated vascular endothelial growth factor expression under hypoxic condition

**Figure 4:**
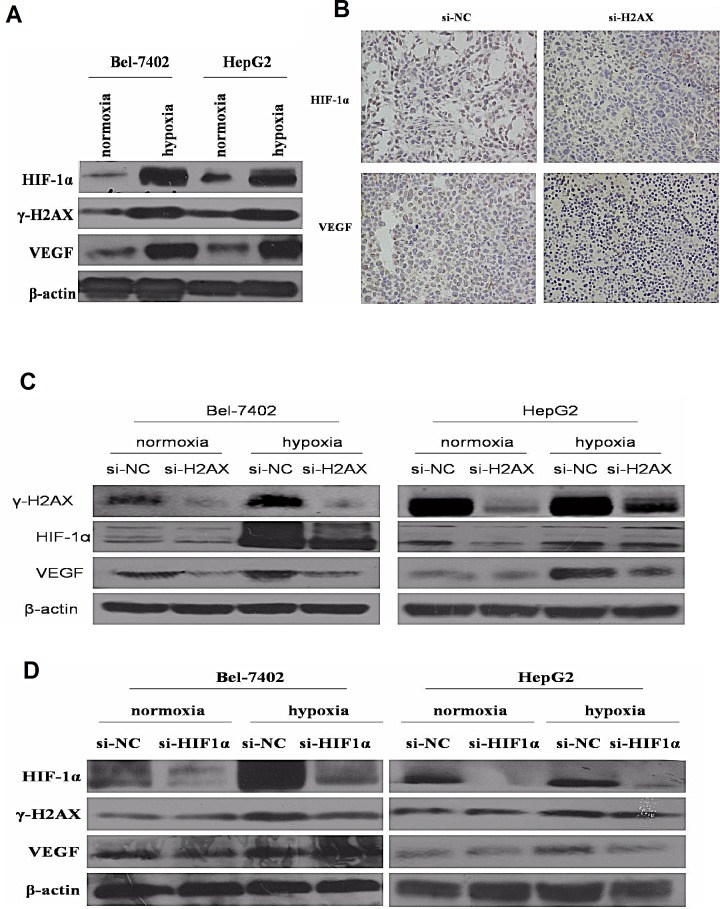
γ-H2AX is essential for HIF-1α-mediated VEGF expression (A) Effects of hypoxia on HIF-1α, γ-H2AX and VEGF expression. (B) Immunohistochemistry analysis of HIF-1α and VEGF expression *in vivo* tumorigenesis assay. (C) Expression of HIF-1α and VEGF in HCC cell lines transfected with H2AX siRNA or Con siRNA under normoxic or hypoxic conditions. (D) Expression of γ-H2AX and VEGF in HCC cell lines transfected with HIF-1α siRNA or Con siRNA under normoxic or hypoxic conditions.

Recently, some studies have suggested that in the knock down of H2AX under hypoxic conditions, the hypoxia-driven retina angiogenesis was obviously weakened [14-16]. Thus, we questioned whether γ-H2AX regulates HIF-1α activity under hypoxic condition in HCC. Both γ-H2AX and HIF-1α expression were increased under hypoxic conditions (Fig. 4A). Moreover, decreased HIF-1α level accompanied with VEGF was also observed in H2AX knockdown-injected tumors (Fig. 4B). As shown in Fig. 4C, H2AX knock-down in Bel-7402 or HepG2 cells resulted in down-regulation of HIF-1α and VEGF expressions under normoxic or hypoxic conditions. HIF-a siRNA significantly inhibited HIF-1α protein expression level but not γ-H2AX level (Fig. 4D). These results suggested that γ-H2AX and γ-H2AX may promote angiogenesis of HCC through HIF-1α.

### The EGFR pathway plays a critical role in mediating γ-H2AX function under hypoxic condition

**Figure 5:**
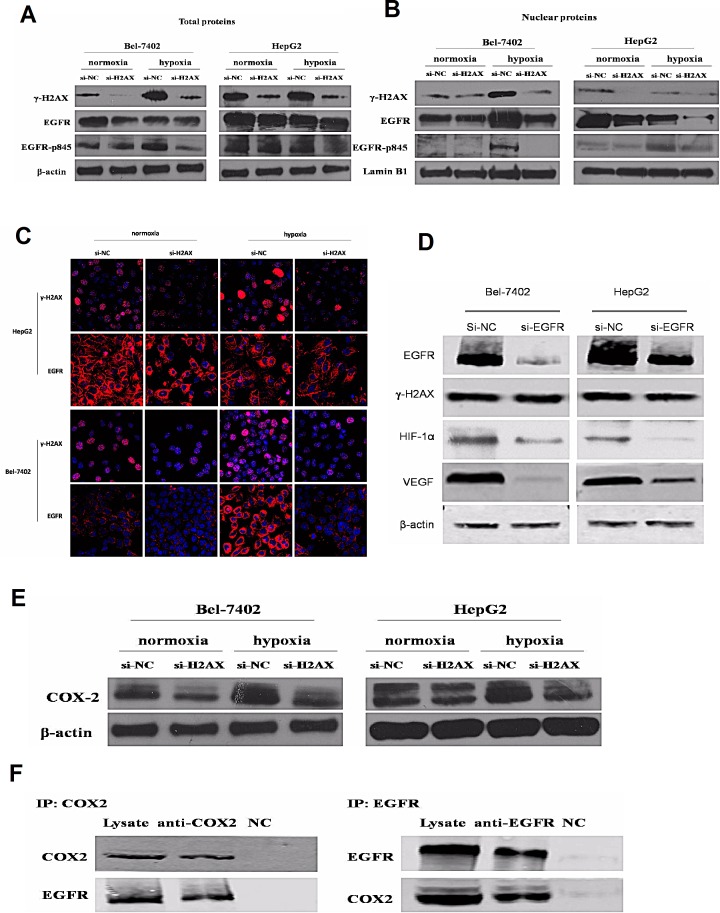
γ-H2AX regulates VEGF and HIF-1α correlated with COX2/EGFR (A) Effects of H2AX knockdown on EGFR and p-EGFR were measured by western blot. (B) Effects of γ-H2AX on nuclear EGFR protein expression were measured by western blot and immunofluorescence. (D) Effects of EGFR knockdown on HIF-1α, γ-H2AX and VEGF expression. (E) Effects of γ-H2AX down-regulation on COX2 expression. (F) Immunoblot analysis of the interaction between EGFR and COX2 by co-immunoprecipitation.

Previous studies suggest that EGFR protein combines with γ-H2AX under ionizing radiation [24]. EGFR signaling pathway was analyzed by expression of EGFR, phosphorylated forms of EGFR. The total protein expression of EGFR and EGFR-p845 was significantly inhibited in H2AX knock-down HCC cell lines under normoxic or hypoxic conditions, whereas H2AX knock-down could effectively inhibited the EGFR translocating into the cell nucleus (Fig. 5A, B, C). Moreover, EGFR siRNA significantly inhibited EGFR, HIF-1α and VEGF protein expression levels but not γ-H2AX level (Fig. 5D). These results suggest the role of EGFR during γ-H2AX regulated angiogenesis of HCC, and imply a model of γ-H2AX activation of EGFR-HIF-1α-VEGF signaling that results in angiogenesis of HCC cells.

Through immunoblot, we detected the COX-2 expression; its protein level was significantly decreased after the knock-down of H2AX expression (Fig. 5E). To detect the functional relevance of interaction between EGFR and COX2, we performed EGFR/COX2 co-immunoprecipitation. We indeed observed COX2/EGFR in complex with EGFR/COX2 (Fig. 5F). These results suggest a mechanism in which γ-H2AX activates EGFR through control of COX-2 expression.

### Combined γ-H2AX, HIF-1α and EGFR has better prognostic value for HCC after liver transplantation

**Figure 6:**
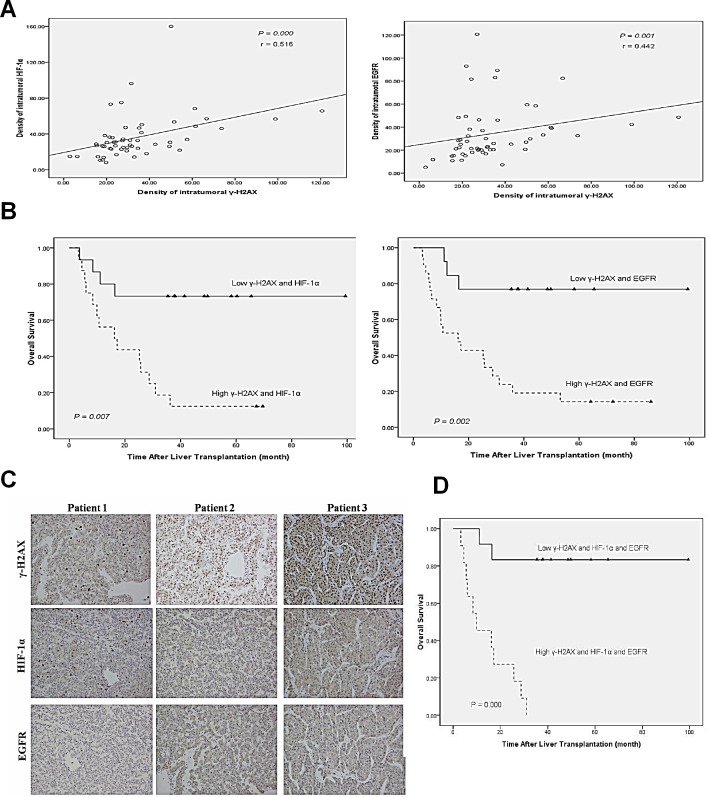
Combined γ-H2AX, HIF-1α and EGFR has better prognostic value for HCC after LT (A) Correlation between γ-H2AX expression and HIF-1α/EGFR level examined in tumor tissues derived from 57 patients. (B) Combination of γ-H2AX expression and HIF-1α/EGFR significantly enhanced correlation in the over-all survival rate. (C) Representative immunostaining for γ-H2AX, HIF-1α and EGFR is shown for three patient samples. (D) Combination of γ-H2AX, HIF-1α and EGFR enhanced correlation to the significance for poor prognosis.

**Figure 7:**
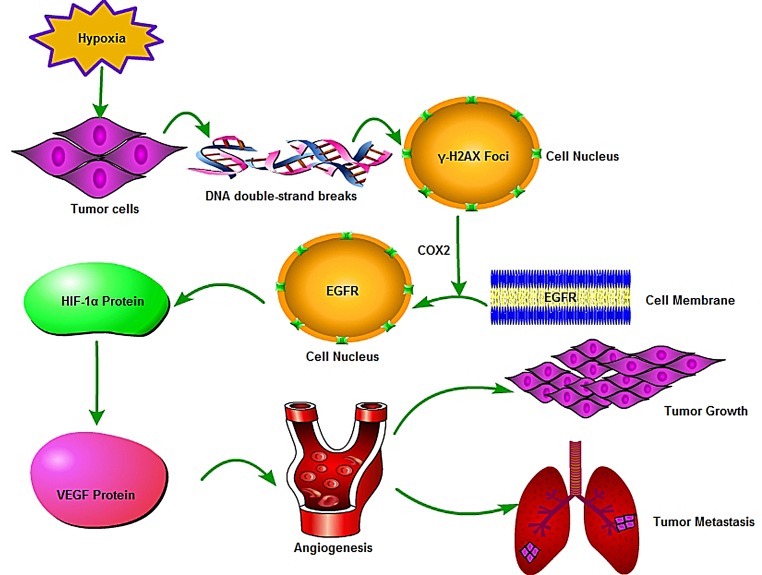
A model for γ-H2AX action in HCC angiogenesis via EGFR/HIF-1α/VEGF signaling pathway This signaling pathway was drawn by the Pathway Builder Tool 2.0. Hypoxia can induce the expression of γ-H2AX via cleavage of the DNA double-strand. The induced γ-H2AX could promote EGFR to translocate into the cell nucleus from the cell membrane through regulate COX2 expression. Nuclear localized EGFR was found to induce HIF-1α expression which resulted in VEGF expression and angiogenesis.

We further analyzed the expression levels of γ-H2AX, HIF-1α and EGFR in 57 clinical HCC samples by immunohistochemistry. Tissue microarray analysis revealed a correlation between γ-H2AX with HIF-1α (r = 0.516, p = 0.000), and with EGFR (r = 0.442, p = 0.001) (Fig. 6A). For patients whose tumor had high levels of both γ-H2AX and HIF-1α had worse over-all survival, so as with high levels of both γ-H2AXand EGFR (Fig. 6B, C). Using a combination of these three markers increased the prognostic value, as compared to a combination of high levels of γ-H2AX and HIF1α/EGFR (Fig. 6D). In sum, evaluation of γ-H2AX, EGFR, HIF-1α expression is a powerful predictor of poor prognosis after LT, further supporting a model of γ-H2AX activation of EGFR-HIF-1α-VEGF signaling (Fig. 7) resulting in the angiogenesis of HCC cells.

## DISCUSSION

The endogenous level of γ-H2AX was highly expressed in many premalignant lesions, cancer cells and solid tumors [25-27]. However, little is known about the role of γ-H2AX in HCC. The findings of this study are; 1) the study showed high expression of γ-H2AX in tumor tissues, compared to the peritumoral tissues, as well as normal tissues. 2) the clinical data suggests that γ-H2AX significantly correlates with tumor size and vascular invasion, and HCC tissues with high γ-H2AX expression has worse overall or tumor-free survival rate than low expression. 3) γ-H2AX expression directly regulates the angiogenesis and tumorigenicity of HCC cell lines *in vivo*. All in all, these results provide the first evidence supporting the pro-oncogenic and pro-angiogenesis function of γ-H2AX in HCC.

In this study, we further investigated the mechanism of γ-H2AX on the angiogenic activity of HCC. We observed that γ-H2AX down-regulation inhibits VEGF expression and the formation of capillary-like tubes. We also discovered that decrease in HIF-1α, EGFR and COX2 expressions were involved in the angiogenesis suppression of VEGF expression by γ-H2AX down-regulation.

Previous studies suggest that hypoxia can induce the expression of γ-H2AX through the DNA damage response [9, 13]. Further, knockdown of HIF-1α or HIF-2α could delay the γ-H2AX accumulation, but not decrease γ-H2AX expression further under hypoxic conditions [13]. These reports are in good agreement with our study. To detect a relationship between γ-H2AX and HIF-1α, we pre-treated HCC cell lines with siRNAs targeting H2AX and then cultured the cell under hypoxic condition. We found that both γ-H2AX and HIF-1α were inhibited under normoxic or hypoxic conditions, similar to VEGF expression. This observation suggests the notion that hypoxia-induced γ-H2AX foci have an important role in hypoxia induced angiogenesis through HIF-1α/VEGF pathway.

EGFR activation has been shown to induce HIF-1α expression and to led to HIF-1α up-regulation [28, 29]. Consistent with these reports, our results show that HIF-1α and VEGF expression was down-regulated after EGFR was knockdown by siRNAs while γ-H2AX expression did not change. Contrary to one report that the reduction of nuclear EGFR protein could increase the amount of γ-H2AX after irradiation [24]. Some reports have suggested that EGFR protein combines with γ-H2AX under ionizing radiation [24, 30]. According to our study, we found that γ-H2AX down-regulation could effectively suppress the EGFR expression under normoxic or hypoxic conditions. Additionally, nuclear-localized EGFR is highly associated with many biological processes such as proliferation and angiogenesis [31], we were able to show that nuclear-localized EGFR was significantly suppressed after γ-H2AX down-regulation. We also found that COX2 protein level was significantly decreased after knock-down H2AX expression, and EGFR and COX2 are interactive proteins, this results are consistent with the previous study which reports that COX2 inhibitor could suppress the expression of nuclear localized EGFR [32]. These results again suggest the notion that γ-H2AX activation of EGFR-HIF-1α-VEGF signaling pathway results in angiogenesis of HCC.

Remarkably, the predictive value of γ-H2AX expression levels combined with EGFR and HIF-1α signal was more sensitive than that of γ-H2AX alone for over-all survival rate. This result suggests that the identification of tumor γ-H2AX alone or combined evaluation of γ-H2AX/EGFR/HIF-1α levels as a new prognostic marker in patients with HCC after LT. This is important because they provide not only a new criterion for prognosis, but also a potential therapeutic target. DNA damage response exists in therapies such as transcatheter arterial chemoembolization (TACE) and Stereotactic body radiotherapy (SBRT), and could lead to death of tumor cells. However, as a result of DNA damage response, the elevatory expression of γ-H2AX could also promote the angiogenesis of HCC cells. Therefore, inhibitors of γ-H2AX will be helpful in improving the efficacy of TACE and SBRT. It was recently reported that rapamycin and other rapalogs could effectively reduce the generating of γ-H2AX [33, 34]. So, there are brighter prospects for application of rapamycin in treatment of HCC.

Altogether, we demonstrated that γ-H2AX is associated with angiogenesis of HCC cells through EGFR/HIF-1α/VEGF signaling pathway. Also, this study suggests that γ-H2AX or combination of γ-H2AX/EGFR/HIF-1α is a novel marker in the prognosis of HCC after LT and a potential therapeutic target.

## MATERIAL AND METHODS

### Clinical specimen collection

Fifty-seven patients receiving orthotopic liver transplantation (OLT) for HCC at our hospital (First Affiliated Hospital, Zhejiang University School of Medicine, Zhejiang, China ) between 2005 and 2010, 37 peritumoral liver tissues and 17 normal liver tissues were collected in this study. Letter of consent was obtained from all patients, and the experimental protocols were approved by the local ethics committee.

### Immunohistochemistry

Immunohistochemistry was performed as described previously [35]. The tissue arrays were deparaffinized in xylene and rehydrated in a series of graded alcohol dilutions. Heat epitope retrieval was done for 20 minutes in target-retrieval solution at pH7.5. Sections were incubated with a rabbit monoclonal antibody to human γ-H2AX (EGFR, HIF-1α) at dilution of 1:250 for overnight at 4C°. Slides were then incubated with HRP at room temperature for 30 minutes and was visualized using DAB as chromogen for 5-10 minutes. Slides were counterstained with hematoxylin. Meanwhile, the immunoreactive density was assessed by two independent investigators in our team without prior knowledge of clinical pathologic data by the software Imagepro Plus 6.0 software. For the reading of antibody staining, a uniform setting for all the slides was applied. Integrated optical density of all the positive staining in each photograph was measured, and its ratio to total area of each photograph was calculated as density. The color of immunoreactive density was chosen by histogram based, and false positive area was wiped out by Filter option, the assessed data were used to draw column diagram. Paraffin sections were scored semiquantitatively as follows: Grade 0: 0% immunoreactive cells; Grade 1: ≦5% immunoreactive cells; Grade 2: >5-50% immunoreactive cells; Grade 3: ≧50 immunoreactive cells. For statistical purposes, cases with Grade 0 and 1 were considered as low expression, those with Grade 2 and 3 were considered as high expression.

### Cell culture

Nine human HCC cell lines (HepG2, Hep3B, Huh-7, Bel-7402, SK-Hep-1, SMMC-7721, MHCC-97L, MHCC-97H and MHCC-LM3), one immortalized liver cell lines (L-02) and human umbilical vein endothelial cells(HUVECs) were purchased from Cell Bank of Type Culture Collection of Chinese Academy of Sciences, Shanghai Institute of Cell Biology, Chinese Academy of Sciences and were cultivated as described by the suppliers. These cells were obtained directly from a cell bank that performs cell line characterizations and passages in our laboratory for less than six months after receipt or resuscitation. HUVEC were used in low passages (up to the sixth passage).

### mRNA microarray analysis

Microarray profiles were obtained using a Human U133 plus 2.0 (Affymetrix Technologies), and used to identify mRNA differentially expressed in normoxic and hypoxic cells. 3-fold increase and 2-fold decrease in hypoxic cells were selected for significant differential expression.

### Antibodies

Antibodies recognizing H2AX (ab11175), phospho-H2AX(Ser139) (ab2893), VEGF-A (ab183100), HIF-1α (ab1), EGFR (ab2430) and COX2 (ab15191) were obtained from Abcam Company(U.S).

### Transfections

HepG2, Bel-7402 were transfected with specific siRNA targeting H2AX and HIF-1α, EGFR mRNA or a shRNA(Shanghai GeneChem Co., Ltd., Shanghai, China). Human H2AX siRNA (5′-CACCCAGGCCUCCCAGGAGUACUAA-3′); Human HIF-1α siRNA (5′-GAAAUUCCUUUAGAUAGCAAGACUU-3′); Human EGFR siRNA (5′-CGGAAUAGGUAUUGGUGAAUUUAAA-3′). For cell transfection, 5×10^3^cells/cm^2^ were plated in six-well plates. Cells were transfected with a final siRNA concentration of 100nM using transfectin (Bio-Rad laboratories, Inc, Hercules CA, USA).

### Western blot

As described previous [36], non-transfected cells or cells transfected with different siRNAs were subjected to the following treatments: no treatment (normoxia), hypoxia (O_2_ of 1%) in a hypoxia chamber for 72 hours. Soluble proteins were collected and stored at −80°C after a centrifugation at 12000g for 15min. Protein amount was determined by Bradford assay (Bio-Rad). For testing, after denaturation, the proteins were separated with gel electrophoresis using 10% SDS-PAGE and then wet transferred to PVDF membrane for 2 hours of blocking in 5% skim milk. The membrane was washed once with TBST, then incubated overnight at 4°C with relevant antibodies(1:1000). The membrane was again washed three times with TBST, then incubated with secondary antibody (Goat Anti-Rabbit/mouse IgG 1:2000) for 2h at room temperature. The membrane was washed the third time with TBST and then ECL liquid was added, and placed to react in a darkroom. β-actin and GAPDH (1/2000) was used as a positive control.

### Immunofluorescence

Immunofluorescence was used to observe γ-H2AX and EGFR expresssion. Cells were seeded into confocal dishes containing a sterile coverslip on the bottom. At 48 h after transfection with H2AX siRNA, cells were washed with PBS, incubated with Hoechst 33342 (Invitrogen, USA) for 15 min, fixed using 2% paraformaldehyde for 5 min, permeabilised using Triton X-100 0.1% for 10 min, and blocked using 10% FBS for 1 h at room temperature. Next, the cells were incubated with a 1:500 dilution of anti-γ-H2AX monoclonal antibody or anti-EGFR monoclonal antibody (Abcam, USA) overnight at 4°C. All samples were washed twice in PBS and incubated with goat-anti-rabbit CY3 as the secondary antibody (Beyotime Institute of Biotechnology, China). Finally, samples were washed twice in PBS and examined by confocal microscopy (Olympus FV1000, Japan) to produce a merged image.

### Co-immunoprecipitation

Dynabeads^®^ Co-Immunoprecipitation Kit (Life technologies, Catalog number 14321D) was used to perform co-immunoprecipitation and the process was according to the user guide of this Kit. To extract protein, HepG2 cells was resuspended in Extraction Buffer(1X IP ; 100 mM NaCl ; 2mM MgCl2 ; 1mM DTT) on ice for 15 min. The cell lysate was centrifuged (2600 g, 5 min) at 4 °C and the supernatant was collected. For every kinds of antibody, 1.5mg beads and 10 μg antibody were incubated at 37°C for 24 hours. HB wash buffer (added with 0.1% Tween®-20), LB wash buffer (added with 0.1% Tween-20), SB wash buffer and Extraction Buffer was used in turn to wash the antibody-coupled beads. For every kinds of antibody-coupled beads,1.5 mg beads were resuspended in 1 ml cell lysate at 4 °C for 30 min. Washed by Extraction Buffer and Last Wash Buffer (1X LWB ; 0.02% Tween®-20), the beads were then resuspended in 60 μl Elution Buffer and incubated at room temperature for 5 min. The supernatant of elute liquid was collected on a magnet. The elute liquid contains the purified protein complex. Western blot was performed to analyze the proteins in the cell lysate and elute.

### *In vitro* tube formation assay and quantification of angiogenesis *in vivo*

Non-transfected tumor cells or cells transfected with H2AX siRNA were subjected to the following treatments: Transfected 24 hours later, tumor cells were cultured with serum-free medium for 24 hours. After incubation, the tumor cells supernatant was collected and centrifuged at 3000 r/min for 15 min to remove dead cells and other impurities. The supernatant was to be used as conditioned medium. 5×10^4^ HUVECs were plated into the Matrigel coated wells and incubated at 37°C in the conditioned medium. After incubating after 16 hours, the plates were photographed. Tube formation was quantified by counting the number of connected cells in five random fields. To analysis of tumor angiogenesis *in vivo*, tumor sections were stained with an antibody against CD34. Regions of vascular density within the tumors were examined. The number of CD34-positive area per microscopic field was recorded.

### Tumor xenograft experiments

All experimental procedures were performed in accordance with the National Institute Guide for the care and use of laboratory animals. 1×10^7^ cells were resuspended in 100μl PBS and injected subcutaneously into the lateral flanks of immunodeficient mice. Tumors volumes were measured every third day after one week, using the equation: V (cm^3^) = width^2^ (cm^2^) × length (cm) /2. Tumors were harvested for immunostaining four weeks later after tumor implantation.

### Statistical analysis

SPSS17.0 software was used for statistical analysis. The experimental data were expressed as mean ± SD, and assessed by a two-tailed Student T test. Over-all survival rate was calculated with the Kaplan-Meier method, and the statistical difference between survival curves was determined with the log-rank test. Statistical significance was accepted if *P*<0.05.
